# Impacts of an anxiolytic drug on fish behaviour and habitat use in a natural landscape

**DOI:** 10.1098/rspb.2025.1443

**Published:** 2025-09-10

**Authors:** Natalia Sandoval Herrera, Erin S. McCallum, Henrik Baktoft, Christer Brönmark, Daniel Cerveny, Lars-Anders Hansson, Gustav Hellström, Kaj Hulthén, P. Anders Nilsson, Tomas Brodin

**Affiliations:** ^1^ Department of Wildlife, Fish and Environmental Studies, Swedish University of Agricultural Sciences, 901 83 Umeå, Västerbotten County, Sweden; ^2^ National Institute of Aquatic Resources, Danish Technical University, 8600 Silkeborg, Denmark; ^3^ Aquatic Ecology Unit, Department of Biology, Lund University, Lund 223 62, Sweden; ^4^ Faculty of Fisheries and Protection of Waters, South Bohemian Research Center of Aquaculture and Biodiversity of Hydrocenoses, University of South Bohemia in Ceske Budejovice, 389 25 Vodnany, Czech Republic

**Keywords:** predation, benzodiazepine, telemetry, home range, oxazepam, fish, behaviour

## Abstract

Pharmaceutical contaminants reaching natural aquatic ecosystems can affect fish behaviour, modifying activity patterns, foraging behaviour and antipredator responses. While laboratory-based studies can offer key insights, assessing the ecological relevance of these findings requires field-based approaches. Therefore, we examined the effects of oxazepam, a widely prescribed anxiolytic drug, on the behaviour of a cyprinid fish (the common roach, *Rutilus rutilus*) in the wild, combining slow-release exposure implants with continuous tracking via acoustic telemetry. To add ecological realism, we created a landscape of fear with an uneven distribution of resources (macrophytes) and exposure to predators (pike, *Esox lucius*), additionally testing the effects of the drug on roach habitat selection and predator–prey interactions. Fish exposed to the drug showed an increased swimming activity and speed, but exhibited a more constrained spatial distribution in the pond, favouring areas with higher refuge availability. Both exposed and unexposed fish modified their habitat use in the presence of predators. Exposed fish appeared to get closer to the predators when these were caged, but not when predators were free-roaming. Our findings highlight the importance of considering ecological context to understand how pharmaceuticals affect fish behaviour, which is crucial for assessing risks at population and ecosystem levels.

## 1. Introduction

Pharmaceutical contamination of aquatic ecosystems is a growing concern worldwide. As human consumption of, for example, anxiolytic drugs continues to increase, the discharge into the environment, and especially to aquatic ecosystems through wastewater, has become a significant issue [1–3]. These compounds, designed to improve human and animal health, pose unintended consequences for non-target organisms inhabiting aquatic systems (reviewed in [4,5]). Among pharmaceuticals, anxiolytics (used to treat anxiety) are an increasingly prescribed medication found in aquatic ecosystems worldwide [1,6]. These neuroactive drugs are designed to modify human behaviour but can also alter fish behaviour and physiology [4]. In fish, these behavioural effects include modification of activity patterns [7], foraging behaviour [8], predator–prey interactions [9,10] and social behaviour [11,12]. Such alterations can potentially lead to cascading effects on population dynamics, community interactions and overall ecosystem functioning [13–15].

While laboratory-based studies have provided important initial insights into the potential effects of neuroactive pharmaceuticals on fish behaviour, they poorly represent the complexity of natural settings, characterized by fluctuations in weather, resource availability and predation risk [16]. Research conducted in natural field settings, which has been limited to date, is crucial for assessing the ecological relevance of previous laboratory-based findings. Experiments in the wild allow study organisms to express their full behavioural repertoires unconstrained by the spatial and temporal limitations of tank experiments, and, further, testing whether changes in individual behaviour may have effects at higher levels of biological organization [17]. The limited number of such studies arises partly from the logistic constraints associated with studying behaviour in the wild in aquatic environments. Recent advances in behavioural ecology and ecotoxicology, however, offer promising solutions to these longstanding limitations [18]. For instance, animal-tracking technologies like acoustic telemetry now allow us to monitor individual animal movements with high accuracy underwater [19,20]. High-resolution telemetry offers an even more detailed recording of fish movements at fine spatial and temporal scales (e.g. positions every second), providing valuable insights into how individuals interact with their environment and respond to anthropogenic changes [21–23]. Moreover, the use of slow-release implants, which allows continuous and controlled exposure of focal individuals without the need for whole-ecosystem manipulations, facilitates the creation of realistic exposure scenarios in the field, altogether enhancing the ecological relevance of toxicant exposure studies [24,25]. Combining these technical advances thus provides an integrative approach that enables us to delve deeper into intra- and interspecific interactions at a temporal and spatial scale relevant for natural conditions.

From the wide array of behaviours that can be studied with this approach, predator–prey interactions are among the most crucial behaviours directly influencing individual survival, thereby shaping community structure and ecosystem functioning. These interactions can be affected by exposure to neuroactive pharmaceuticals such as antidepressants or anxiolytics [9,26–29]. For instance, these drugs may reduce prey vigilance, alter foraging patterns or impair predator recognition [12]. These behavioural disruptions could modify an animal’s perception of predation risk in complex habitats, often referred to as the ‘landscape of fear’ [30]. While the concept of the landscape of fear has been extensively used in ecology to describe prey spatial and temporal distribution [31–34], its application in ecotoxicological studies remains unexplored. Incorporating the landscape of fear framework into field experiments can offer key insights into the direct effects of pollutants on individuals, as well as their indirect impacts on predator–prey interactions and overall ecosystem dynamics.

Here, we aim to bridge the gap between laboratory findings and real-world conditions by integrating several novel techniques to advance our understanding of the ecological impacts of pharmaceutical pollutants in natural ecosystems. Specifically, we have studied how exposure to an anxiolytic pharmaceutical affects fish behaviour in a natural pond environment with spatial heterogeneity in both perceived predation risk and resource availability. We tested how oxazepam, a widely prescribed benzodiazepine medication, affected common roach (*Rutilus rutilus*), a widespread freshwater fish, in the presence of one of its natural predators, the northern pike (*Esox luscius*). We exposed roach to an environmentally realistic dose of oxazepam, or a sham control treatment, using slow-release internal implants [24], and subsequently tracked fish movements within the same pond using high-resolution acoustic telemetry [22]. We then compared activity levels between exposed/unexposed fish, leveraging computational advancements made in the field of animal movement [35] to estimate home ranges, habitat selection and predator–prey interactions—all under a landscape of fear framework where we manipulated the presence and movement of predators over the experiment’s duration. If the presence of predator cues and/or free-roaming predators created a landscape of fear in the pond, we expected to see changes in roach behaviour across predator treatment conditions and that roach should shift their behaviour to avoid predator encounters. Oxazepam induces behavioural changes by enhancing the action of γ-aminobutyric acid (GABA), an inhibitory neurotransmitter that reduces nerve activity. Since GABA receptors are conserved across vertebrates [36,37], we expect oxazepam to affect fish behaviour similarly to mammals. Based on this mode of action and previous studies, we predict oxazepam’s anxiolytic effects in roach should: (i) reduce swimming activity; (ii) reduce predator avoidance by increasing boldness; and (iii) modify home range size and resource use owing to changes in risk perception.

## 2. Material and methods

### (a) Field site: description and preparation of the experimental pond

**Figure 1.**
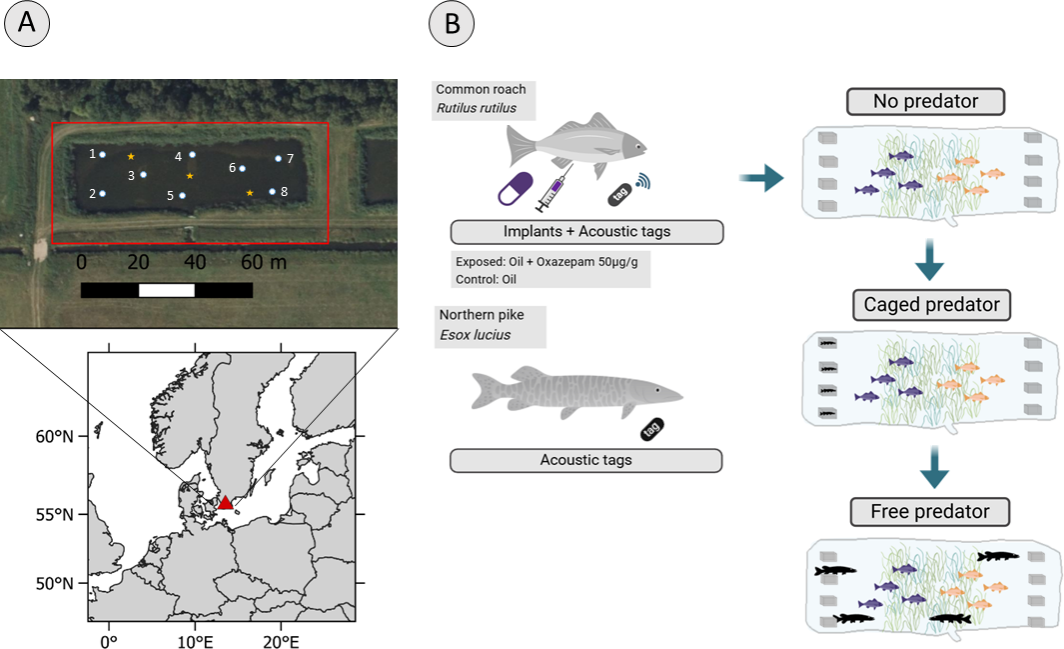
(A) Location and aerial image of the iPonds facility in southern Sweden. White circles represent the position of the receivers in the pond, numbered from 1 to 8, and yellow stars the positions of the reference tags. (B) Schematic representation of the experimental approach and sequence of the predator threat condition.

We conducted this study between 25 October and 7 November 2018 at an experimental pond infrastructure (also known as ‘iPonds’) located in Vomb, Sweden (figure 1A). This area hosts a series of infiltration ponds of similar size (*ca* 90 × 30 m, *ca* 1.2 m deep; see [20] for further details). We used one of these ponds in our study. The pond has a sandy substrate and is usually covered by dense stands of submerged vegetation (mainly *Chara* spp*.*). The pond is supplied with water from nearby Lake Häljasjön (55°40′ N, 13°33′ E) via a small stream running parallel to the pond. The pond has an adjustable inlet (but no outlet) that provided us with the ability to regulate the water level. Before this experiment began, we drained the pond to approximately 30 cm depth and then manually removed the submerged vegetation from the ends of the pond, leaving a patch in the middle as a shelter and food hub (figure 1A). At both ends of the pond, we placed four perforated plastic cages (40 × 60 × 35 cm) that would later hold pike predators before their release into the pond. The cages were fixed to the pond bottom before the start of the experiment (figure 1B) to account for the effect of the cages on fish behaviours. Placing the cages in the vegetation-free ends of the pond allowed prey fish to receive both chemical and visual cues of the predators when they were introduced. While the pond was drained, we also removed all fish individuals residing in the pond prior to the experiments, using electrofishing and netting. Finally, we installed a double-layered net across the pond inlet to prevent any fish from moving in or out of the pond. We then refilled the pond to maximum depth (*ca* 1.2 m). The water quality of the pond was measured weekly and remained very stable during the three weeks of the experiment (dissolved oxygen, mg l^−1^ (average ± standard deviation): 11.2 ± 0.4; oxygen saturation, %: 96.7 ± 2.1; temperature, ^o^C: 9.5 ± 0.4; pH: 7.1 ± 0.1).

### (b) Fish collection

We electrofished 57 wild adult roach (*R. rutilus*) from the lake Krankesjön (55°42′ N, 13°28′ E), southern Sweden. We randomly selected 25 focal roach individuals for oxazepam exposure treatment and 25 individuals to be included as unexposed controls. We also exposed seven additional roach to oxazepam to verify the concentrations of oxazepam present in the muscle, brain and implants (details below) over the course of the experiment. These seven fish were kept in a large keep net in the water supply stream running parallel to the pond so they would experience the same environmental conditions, and two fish were euthanized every week. Four wild adult northern pike (*Esox lucius*) were electrofished from Krankesjön and housed for 14 days in a large keep net in one of the other experimental ponds before the start of the experiment.

### (c) Internal pharmaceutical exposure and fish tagging

We exposed roach to the oxazepam or to a control treatment (with no oxazepam) using internal, slow-release implants placed in the intraperitoneal cavity and, at the same time, we tagged the fish with the acoustic transmitters to track their movements in the pond. We prepared the implants following the method described by [24]. Briefly, we dissolved oxazepam (CAS 604-75-1, Merck) in a fat-based carrier, coconut oil (Kung Markatta Virgin Coconut Oil, https://www.kungmarkatta.se/) at a concentration of 50 µg of oxazepam per gram of carrier. This exposure concentration was previously validated for roach and resulted in a tissue concentration of oxazepam similar to that if fish were exposed to wastewater effluents containing oxazepam [24]. Oxazepam was stirred in the liquefied coconut oil for 20 min and then sonicated in an ultrasound bath (Babdelin Sonorex Digitec) at 30°C for 15 min to ensure it was thoroughly mixed. Control implants were prepared in the same way but without the addition of oxazepam. To tag and implant the fish, we first anaesthetized them in a bath of MS-222 (ethyl 3-aminobenzoate methanesulfonate; Merck; at 0.15 g l^−1^) and measured their body mass (Mettler Toledo scales, accurate to 0.01 g). We then made a small surgical incision anterior to the pelvic fins and inserted the acoustic tag in the abdominal cavity. We then injected the implant containing liquified oxazepam or the control implant into the body cavity via the incision using a blunted needle, at a volume of 10 μl of implant per gram body mass. The incision was sutured with one stich (Ethicon EH7149G 4/0). Fish were then allowed to recover in a separate, aerated tank for 1 h.

Roach had an average body length (± s.d.) of 16.8 ± 1.20 cm, and pike 43.6 ± 11.7 cm. All pike were unexposed and therefore received no implants, but they underwent the same sedation and tagging procedure. All pike were tagged with Vemco/Innovasea V9−2x−180k (9 × 27.5 mm, 4.5 g), and all roach were tagged with Vemco/Innovasea V5−2x−180k (5.7 × 12.7 mm, 0.74 g). We tagged a total of 50 roach and 4 pike individuals. Two of the roach were not detected in the array after a few hours in the pond, presumably owing to tag malfunction. Oxazepam implants resulted in an average internal exposure of 14 ng g^−1^ in brain tissue and 7 ng g^−1^ in muscle tissue, which is equivalent to a waterborne exposure of >1 µg l^−1^ [9,38].

### (d) Acoustic telemetry and fish tracking

The pond was equipped with a high-resolution tracking system consisting of eight acoustic receivers (Vemco/Innovasea HR2) deployed in an array with overlapping detection ranges that continuously monitored the tagged fish (for receiver positions see figure 1A). One receiver malfunctioned (no. 7, see figure 1A), thus tracking was based on the remaining seven. The transmitters were programmed to emit ID-coded signals on average every third second (2.5–3.5 s) and provided a detection efficiency of 93 ± 3% (median ± s.e.). Monitoring of the system performance was achieved by three stationary reference tags. System precision was quantified as the deviation of estimated positions from the median position of each reference transmitter. For the three reference transmitters, respectively, 94, 88 and 99% of estimated positions were within 1 m of the median position (electronic supplementary material, figure S1). Positions of tagged fish were estimated by multilateration using the R package *YAPS* (Yet Another Positioning Solver) [37]. Speed of sound was estimated based on water temperatures measured by the acoustic receivers and supplied to *YAPS* as an input data stream. To minimize the impact of noise and false detections on position estimates, we used the default residual distribution in *YAPS* (i.e. a combined Gaussian and scaled *t-*distribution in which scale and proportion of the *t*-distribution are estimated). Prior to track estimation, detection data timestamps from the seven functioning receivers were synchronized using protocol and functions provided by the *YAPS* package. Synchronization of the receiver array was based on detections of internal transmitters in each of the seven functioning receivers, excluding self-detections. In this study, the synchronization error was below 500 µs in 98% of the synchronization transmitter data (electronic supplementary material, figure S2).

### (e) Landscape of fear treatment

To evaluate the effects of the oxazepam treatment on the perceived predation risk of roach, we manipulated the spatio-temporal distribution of the predators. Our experimental design consisted of three sequential predation risk stages, hereafter predator conditions, each lasting for one week: (i) no predator (only roach in the pond), (ii) caged predator (the pike were held in cages at one end of the pond), and (iii) free-swimming predators (the pike were released from their cages to roam freely). At the start of the first week both oxazepam-exposed and control roach were released in the pond 1 h after receiving the implant, in the absence of predators. In the second week, four pike (predators) were introduced into the predator cages on the west side of the pond, one in each of the cages (figure 1B). At the beginning of the third week, the caged pike were set free in the pond and the empty cages remained in place.

### (f) Chemical analyses of oxazepam in tissues

We extracted oxazepam from the muscle and brain tissues, as well as directly from the implants, of in total six of the additional roach kept in the adjacent stream, with two individuals being sampled every week of the exposure. We prepared the tissues following the procedure described in [24]. See details in electronic supplementary material.

### (g) Data analyses and statistics

Our study had two experimental treatments: oxazepam exposure (control fish and oxazepam-exposed fish) and the landscape of fear predation treatment ((i) no predators, (ii) caged predators, and (iii) free-roaming predators; these stages are called conditions throughout). We separated the data into three datasets, one per week, representing the different stages of the predation treatment. Furthermore, we assigned a time of day (day and night) to each detection based on the respective sunrise and sunset times for the time of the study (e.g. range 25 October to 11 November, sunrise: 6.53 UTC + 2; sunset: 16.00 UTC + 2; change to UTC + 1 on 28 October). Notice that nighttime includes twilight, a transition period with impact on fish activity. We also would like to acknowledge that, because of the prompt release of the fish after tagging, we cannot completely exclude tagging effects; however, each treatment was handled the same, meaning these effects would be similar across groups.

#### (i) Data filtering

Even though the positioning data are very accurate, for high-resolution data, there will still be some degree of location error in the positions (discussed in [39]). Therefore, data were filtered, and some positions were removed prior to analysis. We used the following criteria to remove positions: (i) with standard deviation of *x* and *y* position estimates >2 m; (ii) outside the bounding limits of the polygon of the study area. The preprocessing of the data was done using the package *atlastools* in R [40]. We excluded fish with low positions counts to reduce bias in our analyses, focusing only on individuals with at least 50% of the maximum daily positions (over 6000 positions per day) and those successfully positioned on at least 3 of 6 days per week (excluded: week 1, *n* = 8; week 2, *n* = 4; week 3; *n* = 5). Roach fates were inferred based on detection patterns and trajectories throughout the study [41] and classified into four categories: alive (*n* = 45), natural mortality (*n* = 3), tag malfunction (*n* = 2) and pike predation (*n* = 0). We assumed natural mortalities as fish with no movement, or small and erratic movement for the whole week, or disappearance from the array without return (which could indicate non-pike predation by aerial or terrestrial predators). Pike predation assessments were further analysed in-depth, as detailed below.

#### (ii) Swimming activity

We used swimming speed and activity index as metrics of fish activity. We first calculated the activity index, which corresponded to the proportion of positions for which the fish was active (speed >0.02 m s^−1^). We then calculated the swimming speed by applying a continuous time movement framework as described by [42], where we focused exclusively on active movement, filtering out positions with estimated speeds below 0.02 and above 0.2 m s^−1^ range previously reported as biologically meaningful for the species [43]. We used the mean values per individual, per predation condition, and per time of day, except for the first week, where we also evaluated the swimming activity by day, since the drug is metabolized more quickly during the first few days following the exposure [24]. For swimming speed, we fitted a linear mixed model (LMM, estimated using REML and nloptwrap optimizer via the R package *lme4*) with speed as response variable. For the activity index, we used a genralized LMM (GLMM) with a beta distribution. In both models, we included condition ((i) no predator, (ii) caged predator, and (iii) free predator), time of day (day/night) and treatment (oxazepam, control) as predictors, as well as all two-way interactions. We selected the best model using the Akaike information criteria (AICs). We additionally fitted a separate linear mixed effects (LME) model just to week 1 data, when roach were the only species inhabiting the pond, including speed as response variable, and treatment, day and their interactions as predictors. All models included fish ID (tag) as a random effect on the intercept. All model assumptions and diagnostics were assessed via the package *performance* in R.

To evaluate whether exposure to oxazepam affected roach diel activity patterns, we used a cross-correlation analysis of time-series data to compare the hourly mean speed as indicator of roach activity, between oxazepam treatments for each predator condition [44]. This analysis evaluates the synchrony in the periodic pattern of the fish activity; a high correlation coefficient at time lags >0 indicates activity shifts to later hours, at values <0 indicates activity shifts to earlier hours, and at time lag 0 indicates no differences in the diel pattern between treatments. Lastly, we evaluated differences in diel activity in the free-roaming pike (during only the third week) using an LMM with swimming time as response, time of day (day/night) as predictor, and pike ID as random effect.

#### (iii) Home range

We calculated the home range area based on the 95% habitat utilization distribution (UD) for each individual and then used a population-level estimator approach to estimate a mean home range area per treatment and predation condition. We used an autocorrelated kernel density estimation (AKDE) method that accounts for the temporal autocorrelation of high-resolution telemetry data and estimation uncertainty [45]. We then compared the mean home range area between oxazepam treatments for each predation condition separately using the function *meta* from the R package *ctmm* [46]. Briefly, this function estimates population-level average parameters by using individual movement AKDE, while accounting for estimation uncertainties. For this analysis, we report confidence intervals of the ratio between treatments for each condition, with values that include 1 indicating no significant difference between treatments. We complemented this analysis by conducting a separate test where we evaluated the effect of condition and accounted for a repeated measures design. We conducted an LMM with home range area as the response variable, including condition and treatment as fixed effects and fish ID as a random effect.

#### (iv) Habitat selection

To understand the role of landscape features in the habitat selection of roach, we applied a resource selection function (RSF), which estimates the probability of habitat use for each individual fish based on spatial predictors. We included two predictors of roach distribution in the pond (individual UDs): resource availability (macrophyte distribution) and predation risk (predator distribution, weeks 2 and 3). Macrophytes could be seen as valuable resources for foraging and shelter use, and we here mapped the presence of macrophytes using a satellite picture of the pond and manually coding in qGIS whether there were (1) or were not (0) macrophytes in cells of 0.5 × 0.5 m. With this information, we created a raster with the macrophyte distribution. This raster was then used as a predictor in the RSF analysis. For predation risk, we used the distribution of pike: for week 2, we used a buffer area of 1 m around the locations of the pike cages, and for week 3, we used the ‘population level’ predator UD across all four free-roaming pike for the whole week, meaning we used only one UD raster to incorporate predation risk in the RSF model. We present the estimates and confidence intervals of the model parameters corresponding to the resource predictor variables. These selection coefficients indicate attraction when values are positive, indicate avoidance when values are negative, and are considered statistically significant when the confidence intervals do not include 0. We used an enhanced RSF function from the package *ctmm*, which uses a method for autocorrelation-informed likelihood weighting to mitigate autocorrelation and sampling bias issues [47].

#### (v) Behavioural states

To explore behavioural variation of individuals among treatments, we applied a behavioural state estimation method to our 24 h fish-tracking data. We used a nonparametric Bayesian framework called the mixed-membership method for movement (M4), described by [48], available within the *bayesmove* R package. Based on previous descriptions in the animal movement literature, we categorized these behaviours as: encamped, exploration, transiting, and inactive (see details in electronic supplementary material).

Because changes in the proportion of one behavioural state are intrinsically related to changes in the other behaviours, they cannot be evaluated as independent response variables. We used a set of multivariate analyses suitable for this type of compositional data and not sensitive to outliers [49]. We first transformed the time proportions in each behaviour to an isometric log ratio (ILR). We then performed a permutational multivariate analysis of variance (PERMANOVA) using the ILR-transformed variable to evaluate the effect of treatment on the proportion of time in each behaviour. The PERMANOVA was done using the R package *vegan* [50] .

#### (vi) Predator–prey interactions

Predatory–prey interactions could only be analysed when predators were caged or free-roaming, and how the roach interacted with the predators in each of these periods was analysed separately. During the caged predator period (week 2), we measured predator avoidance by calculating each roach’s minimum daily distance to each of the predators. We used a generalized additive mixed model (GAMM) to compare these minimum distances between treatments while testing the effect of time after predator introduction. The model had a gamma distribution, with distance to predator as response variable, treatment as fixed effect, day after predator introduction as smooth term, and roach ID as a random intercept effect.

During the free-predator period (week 3), we analysed predator–prey interactions in three ways. First, we created a proximity distance matrix for each individual roach with each of the predators and used it to estimate the number of potential interactions between the roach and the pike based on an interaction distance threshold of 2 m and both the roach’s and pike’s detections being within >30 s of each other. To test the effect of oxazepam treatment on the summed number of predator interactions, we used a GLM with a negative binomial distribution and a log-link function (package *glmmTMB*). Second, we estimated the risk of predation for each treatment by calculating the encounter rate with the predator, following the approach described in [42]. We obtained a probability of encounter based on the UD of each roach and the pike (all combined). This was followed by the calculation of the conditional distribution of encounter (CDE) for each treatment, which represents graphically the probability of encounter of roach and pike across the pond. Briefly, this is the product of the home range UDs, divided by the coefficient of overlap between them. Third and finally, to detect the occurrence of predation events, we used a dynamic interaction analysis using the R package *WildlifeDI* [51], and visual inspection of animated trajectories of individuals with higher encounter rates. With this analysis, we calculated a dynamic interaction coefficient that indicates, for a given time segment, a measure of cohesiveness in the path of two individuals using the proximity, direction and speed of their movement.

During the free-predator period (week 3), we analysed predator–prey interactions in three ways. First, we created a proximity distance matrix for each individual roach with each of the predators and used it to estimate the number of potential interactions between the roach and the pike based on an interaction distance threshold of 2 m and both the roach’s and pike’s detections being within >30 s of each other. To test the effect of oxazepam treatment on the summed number of predator interactions, we used a GLM with a negative binomial distribution and a log-link function (package *glmmTMB*). Second, we estimated the risk of predation for each treatment by calculating the encounter rate with the predator, following the approach described in [42]. We obtained a probability of encounter based on the UD of each roach and the pike (all combined). This was followed by the calculation of the conditional distribution of encounter (CDE) for each treatment, which represents graphically the probability of encounter of roach and pike across the pond (figure 6C). Briefly, this is the product of the home range UDs, divided by the coefficient of overlap between them. Third and finally, to detect the occurrence of predation events, we used a dynamic interaction analysis using the R package *WildlifeDI* [51] and visual inspection of animated trajectories of individuals with higher encounter rates. With this analysis, we calculated a dynamic interaction coefficient that indicates, for a given time segment, a measure of cohesiveness in the path of two individuals using the proximity, direction and speed of their movement.

**Figure 2.**
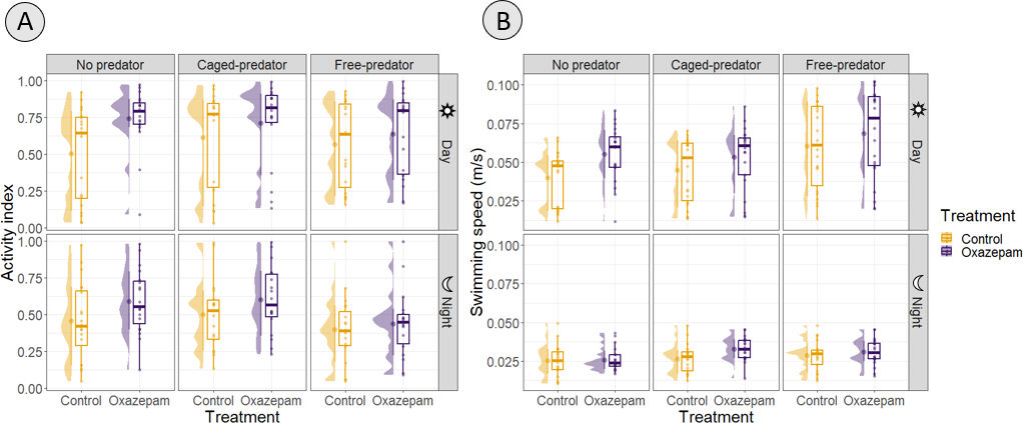
Comparison of weekly swimming activity of roach (*Rutilus rutilus*) during day and nighttime and three experimental conditions: no predator, caged predator and free predator. (A) Median activity index. (B) Median swimming speed. Density plots on the left of the boxplots show the distribution of the data.

## 3. Results

### (a) Swimming activity

We found that roach individuals exposed to oxazepam swam faster than control fish (LMM: *β* = 0.01, *t*(242) = 2.73, *p* = 0.007), regardless of the experimental condition (predator threat) (figure 2; LMM: oxazepam × caged predator: *β* = −1.28e−03, 95% CI [−9.39e−03, 6.84e−03], *t*(242) = −0.31, *p* = 0.757; oxazepam × free predator: *β* = −3.14e−03, *t*(242) = −0.77, *p* = 0.445). The best-performing model was the one that included the two-way interactions between conditions, period and treatment (electronic supplementary material, figure S1). The increase in swimming speed was more evident during daytime (LMM: oxazepam × day: *β* = 0.008, *t*(242) = 2.40, *p* = 0.017) and was as pronounced as 42% in the absence of predators. Roach exposed to oxazepam were less active when the predator was free (GLMM: *β* = −0.65, 95% CI [−1.13, −0.18], *p* = 0.007). As expected from a diurnal species, roach were significantly less active (figure 2A; night: *β* = −0.67, 95% CI [−0.85, −0.48], *p* < .001)) and swam more slowly during nighttime (figure 2B; night: *β* = −0.03, 95% CI [−0.03, −0.02], *t*(247) = −15.12, *p* < 0.001). When assessing how exposure affected roach diel cycles, we found the highest cross-correlation at time lag 0 for all three conditions, meaning that there were no shifts in the activity patterns between treatments. Lastly, both control and oxazepam fish swam faster in the presence of free-roaming predators than in their absence (*β* = 0.02, 95% CI [0.01, 0.02], *t*(242) = 5.16, *p* < 0.001). Concurrently, pike were more active during nighttime (swimming time: *β* = 13854.24, 95% CI [7107.37, 22698.30], *t*(6) = 4.31, *p* = 0.02).

### (b) Home range

**Figure 3.**
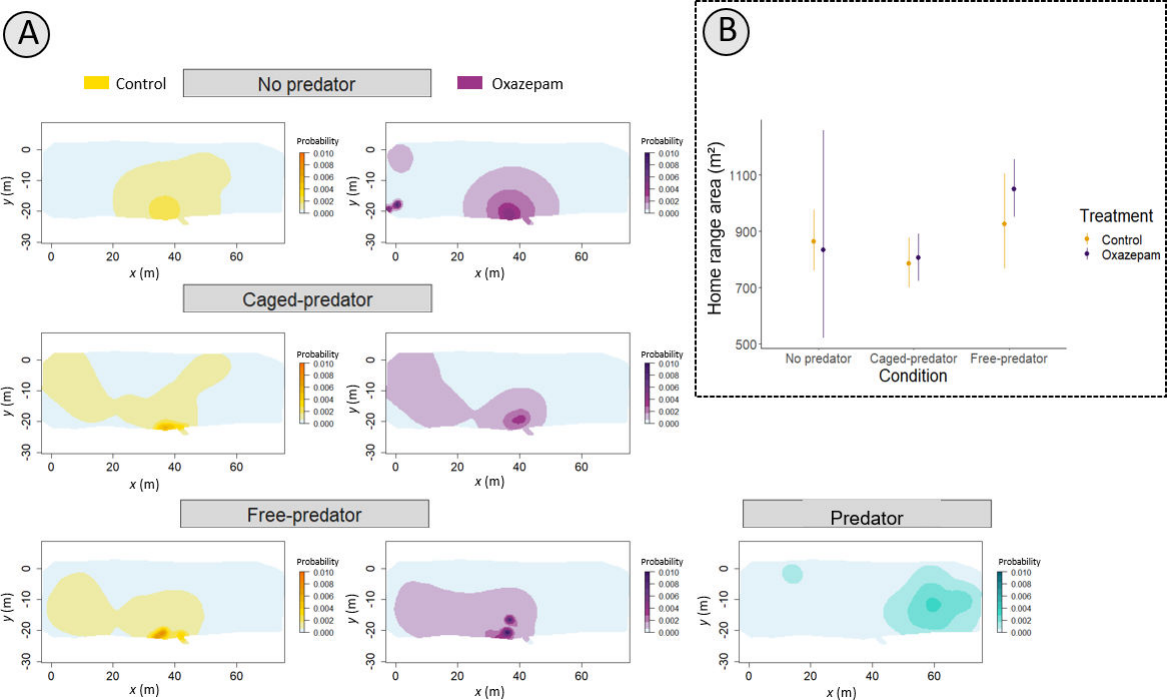
(A) Utilization distribution (probability density) of roach during daytime, averaged across individuals, under different predation threat conditions (no predator, caged predator and free predator). On the bottom right we show the utilization distribution for the free predator. Yellow corresponds to control fish and purple oxazepam-exposed fish. The utilization distribution was obtained using autocorrelated kernel density estimation based on continuous time movement models. More intense colours show core areas of distribution with a higher probability of use. The plot axes correspond to the relative coordinates of the pond (light blue shaded area). (B) Comparison of mean home range size (±95% CI) between treated and untreated roach across predation threat conditions. Mean estimates and confidence intervals were calculated using a *χ*^2^-IG hierarchical model [46]*.*

We found no difference in the home range area between oxazepam treatments for any of the three predator conditions (no predator: ratio = 0.97, CI [0.7, 1.2], *p* = 0.40; caged predator: ratio = 1.01, CI [0.8, 1.4], *p* = 0.29; free predator: ratio = 0.82, CI [0.45, 1.32], *p* = 0.27). Neither oxazepam treatment nor predator condition had a significant effect on the home range area (condition: *F*_102_ = 2.29, *p* = 0.11; treatment: 0.5425; *F*_104_ = 0.4631, *p* = 0.90). As shown in figure 3B, there was an apparent reduction in home range size for both control and oxazepam fish from week 1 (no predator) to week 2 (caged predator), but this difference was not statistically significant. There was a shift in the fish spatial distribution in the pond, where oxazepam-exposed fish used more the areas close to the edge of the pond where the caged predators were located (figure 3A). Control fish, in contrast, seemed to have concentrated their activity in the centre part of the pond. During week 3 (free-roaming predators), as interpreted from the UD maps (figure 3A), both control and exposed fish shifted the core of their home range away from the home range of the predators.

### (c) Habitat selection

**Figure 4.**
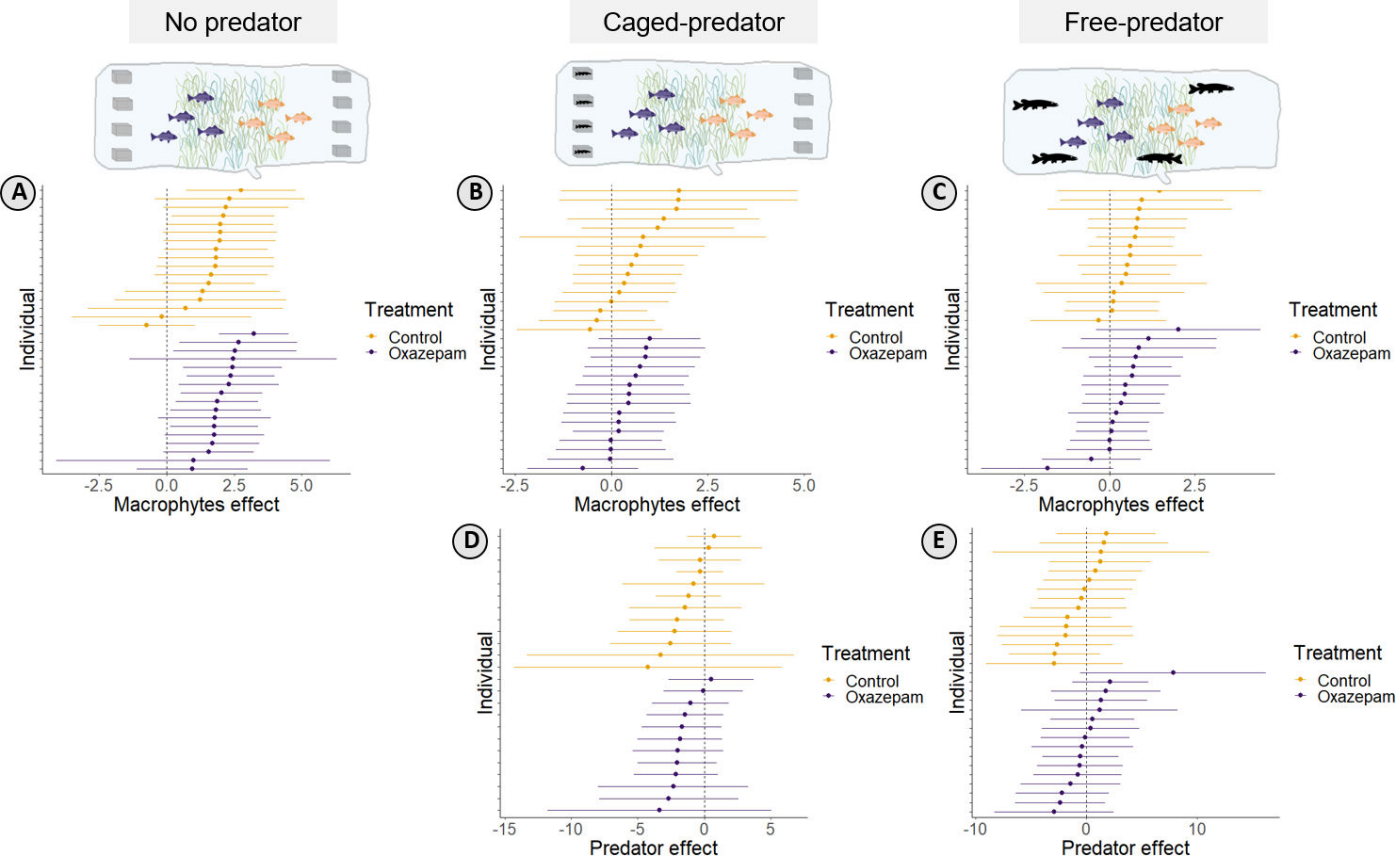
Resource selection coefficients for macrophytes and predator-perceived risk. Effect size is represented as the estimate and 95% confidence interval. Positive values indicate preference and negative values indicate aversion. (A–C) Selection of roach for macrophytes: (A) in the absence of predator, (B) in the presence of caged predator, and (C) in the presence of a free predator. (D,E) Selection (or aversion) of roach for the predators when (D) predators were caged and (E) predators were free.

During the first week (absence of predators), roach selected areas with submerged macrophytes; this effect was most evident for the oxazepam-exposed fish, for which 80% of the individuals had a selection coefficient significantly greater than 0, compared with 17% of control fish (figure 4A). This selection for areas with macrophytes was reduced in the presence of a predator, both when the predators were caged and when they were free-swimming (figure 4B,C), but none of the both positive and negative selection coefficients for macrophytes during these periods was significantly different from 0. As for the effect of the caged predator, for week 2 the selection coefficients suggested that fish avoided the area where the predators were located (figure 4B), although these coefficients were not statistically different from 0. When the predator was free, there was no evident pattern or significant effect in the habitat selection of roach for either treatment (figure 4E).

**Figure 5.**
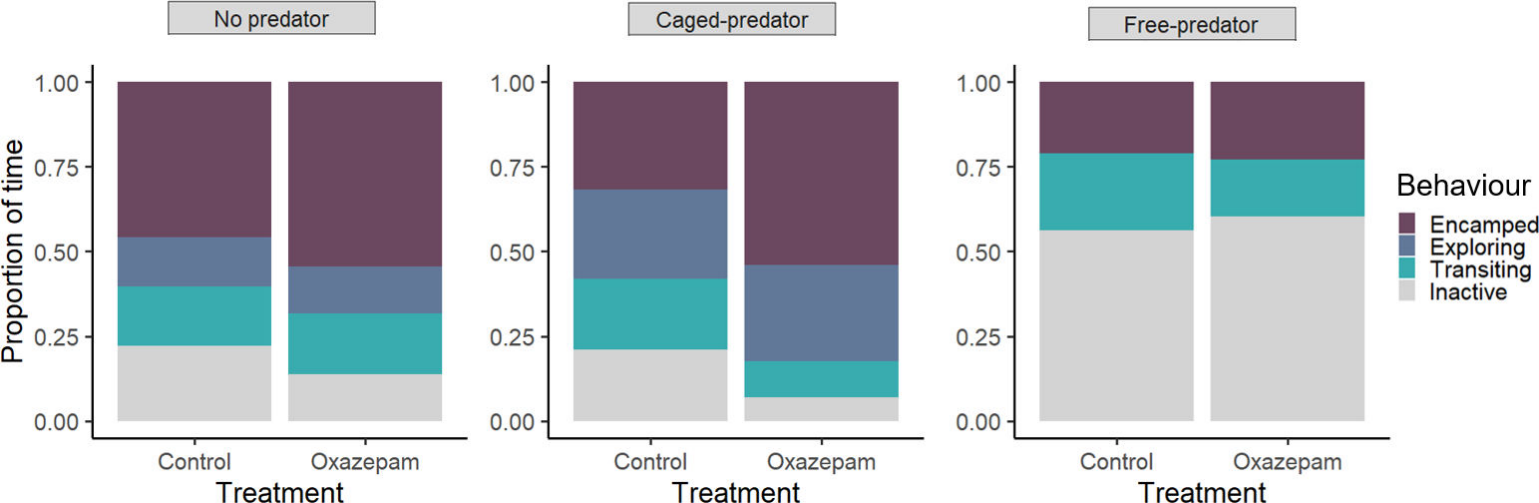
Average proportion of time spent in different behavioural states for roach exposed and unexposed to oxazepam across predation threat conditions.

### (d) Behavioural states

Overall, in a 24 h period fish spent most of their time encamped, and the second most dominant behaviour was exploring. We found significant differences in the activity budget between control and oxazepam-exposed fish in the absence of predators (*F*_2677_ = 32.464; *p* < 0.01); exposed fish spent more time encamped than control fish (figure 5). This behaviour was predominantly observed when the fish were roaming in the middle–southern part of the pond, where the macrophytes were located (visual inspection of tracks). In the presence of caged predators, oxazepam-exposed fish spent almost twice as long in the encamped behaviour than control fish; the differences in the proportions among behaviours were statistically significant (*F*_5797_ = 183.1; *p* < 0.01). Lastly, in the presence of free-swimming predators, both control and exposed fish significantly reduced their time encamped and spent most of their time transiting (*F*_4953_ = 25.047; *p* < 0.01). Notably, under this condition, neither group engaged in exploratory behaviour, which was characterized by lower speeds than transiting, and seemed to spend more than 50% of the time inactive.

### (e) Predator–prey interactions

**Figure 6.**
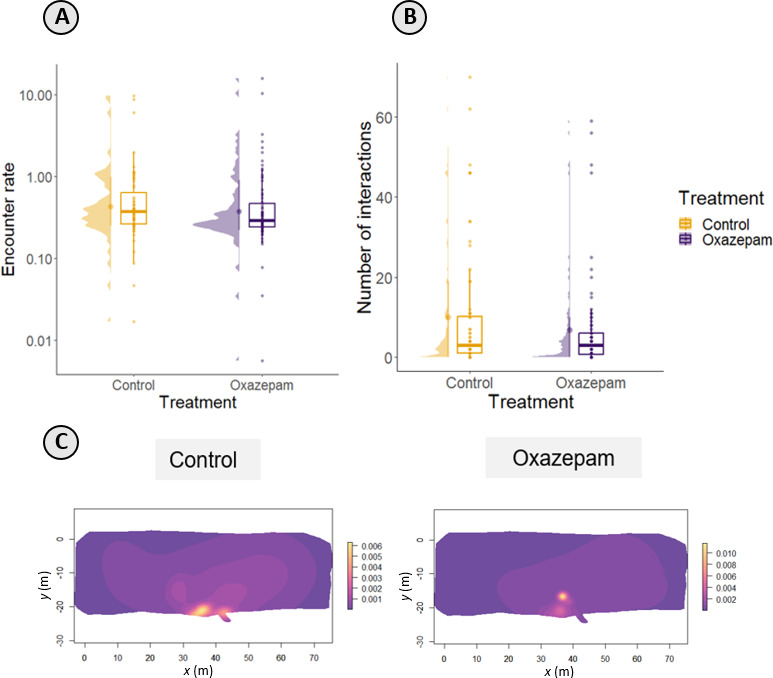
Predator–prey interactions between roach and pike. (A) Median encounter rate probabilities estimation between roach and free-swimming pike; (B) median number of interactions based on proximity to pike; (C) estimated conditional distributions of encounters (CDEs) between roach and free-swimming pike. The lighter colours indicate the encounter locations, which are areas in the pond where the two tracks were <1 m apart.

Oxazepam-exposed fish maintained a shorter distance from the caged predator compared with control fish (GAMM; estimate = 0.008, *t* = 2.27, *p* = 0.02; electronic supplementary material, figure S2), and this proximity did not change across days (*F =* 0.029; *p* = 0.87). When the predator was free-roaming, we did not find differences between oxazepam treatments in the number of interactions with pike as quantified by distance (figure 6B; GLM-negative binomial log-link; estimate = −0.25; *z* = −1.44; *p* = 0.31) nor in the estimated encounter rates based on the overlap of their home ranges (figure 6A; GLM-log-link; estimate = −0.062; *χ*² = 0.32; *p* = 0.57). We did not detect any clear predation events, but we did observe some atypical trajectories that could indicate unsuccessful pike attacks (see electronic supplementary material, figure S3).

## 4. Discussion

Here, we implemented one of the most ecologically relevant experimental designs to date to thoroughly assess the effects of a widespread pharmaceutical pollutant on fish behaviour and ecology. We tested whether exposure to the anxiolytic drug oxazepam would affect the behaviour and perception of predation risk of common roach, using slow-release oxazepam implants, high-resolution acoustic telemetry and a ‘landscape of fear’ approach. This approach allowed us to test our hypotheses of oxazepam effects on swimming activity and boldness under a scenario offering experimental control as well as realistic field conditions. Overall, our findings showed that oxazepam exposure affected individual swimming behaviours and predator–prey interactions under specific predation-risk contexts. Below, we first discuss the overall effect that the ‘landscape of fear’ treatment had on roach in the pond, and then further address the impacts of oxazepam exposure across the different predation-risk contexts.

We created a dynamic landscape of fear with temporal and spatial variation in predation threat by introducing caged predators in week 2 and free-swimming predators in week 3 of our experiment. We used northern pike, one of the most common piscivorous species in northern Europe, and a well known predator of roach [52,53]. We predicted that roach would detect olfactory, as well as visual cues from the pike and would therefore proactively reduce predation risk by avoiding areas in close proximity to the predators. As expected, roach avoided pike by modifying their spatial distribution in the pond and altering their activity times, providing evidence of spatial and temporal landscapes of fear in our experiment. We observed that roach moved away from the free-swimming predators’ home range, indicating a response to perceived predation risk and confirming the existence of a landscape of fear within the pond [31,34]. Moreover, the significant increase in the time of inactivity when the predator was free suggests that roach modified their behaviour to minimize predation risk. Additionally, roach nighttime activity decreased after pike release, aligning with pike’s activity patterns, indicating a temporal response to predation risk, known as the ‘schedule of fear’ [32]. These results align well with previous studies on roach antipredator behaviour, which have shown changes in habitat selection and social behaviours in response to visual and chemical cues of pike [54–57].

Regardless of predation threat condition, we observed that fish exposed to oxazepam exhibited increased swimming speed and increased overall activity as compared with control fish. While various studies have reported oxazepam-induced hyperactivity in fish, including Eurasian perch (*Perca fluviatilis*) [58], the common roach (*R. rutilus*) [59] and Atlantic salmon (*Salmo salar*) [60], the physiological mechanisms behind this effect have not been thoroughly elucidated. This response was contradictory to our original predictions and might seem opposite to the intended pharmacological tranquilizing effect of oxazepam, which increases inhibitory signalling in specific neural circuits. Paradoxically, this heightened inhibition can suppress the activity of inhibitory neurons, leading to a net disinhibition of certain motor pathways and an overall increase in activity levels, causing the fish to swim more actively. Such effects have also been noted in humans, and these contradictory effects have been linked to genetic variations in the GABA receptors [61]. It is therefore possible that differences in the structure and composition of GABA receptors in fish—which have not been extensively studied—account for the seemingly increased likelihood of these effects. Another factor to consider is the exposure concentration because paradoxical effects of anxiolytics are also common at lower doses, with sedation only induced at higher, human-therapeutic doses in mammals [62,63] and fish [11,59,64,65]. Here, the concentration of oxazepam in roach tissues was similar to that of 1 μg l^−1^ waterborne exposure, indicating an environmentally relevant exposure [1,66,67].

When we looked at how oxazepam affected behavioural states, we found that throughout all experimental stages, exposed fish spent more time encamped, swimming around a confined area, specifically within submerged macrophyte patches. Roach are known to favour structured habitats as a protective response to predator cues [55], and these patches may also provide food resources for adult roach, given their primary diet of macroinvertebrates and macrophytes [68]. Staying close to an area that provides protection could be advantageous because it could reduce the risk of predation; however, further implications of this behaviour on social interactions and foraging behaviour are unknown. Future studies should assess whether these behavioural differences induced by oxazepam are large enough to translate into population-wide differences in growth or survival.

Although both the oxazepam exposure and the predation treatments affected roach swimming and space-use behaviours, respectively, we observed an interactive effect of the treatments only during the second week, when the predators were caged. This effect makes sense given that having a stationary predator allows isolation of the behavioural response of the prey, making more evident any treatment effects; the predator behaviour of free-roaming pike in week 3 could mask the effects observed in week 2, especially when it is a fairly small pond with several predators moving around. This altogether suggests that effects of the drug on roach perception of predation risk are only evident under specific contexts, highlighting how increased ecological complexity may challenge our ability to detect direct effects of contaminants in the wild. As we predicted, exposed fish maintained a shorter distance from the caged predators compared with control fish, suggesting lower sensitivity to risk or possible predator inspection behaviour. Predator inspection has previously been reported for roach and can be associated with the modality of the predator cue (i.e. visual versus chemical) that is predominant in a particular environment [55]. In our case, roach may have initially detected the predator through chemical cues (and not visual cues) because of the interference of the cage, which could have prompted this inspection behaviour. Regardless, this result indicates that exposure to oxazepam may impair the fish’s natural predator avoidance behaviour, which could act to increase their risk of predation in natural settings. During the free-predator condition, however, we did not find significant differences in the number of interactions, encounter rates or predation events between treatments. This is consistent with previous landscape of fear studies on other species in semi-natural [69] and natural conditions [70]. Predation in the wild is complex and influenced by several factors, including water turbidity/temperature, predator hunger levels, and predator and prey density. It should also be noted that in our study, all fish were allocated to the same pond, thus allowing social interactions (e.g. group formation) between exposed and unexposed fish individuals, which can explain the relatively similar behavioural responses among treatments regarding e.g. speed, activity and home range. Although not the focus of our current study, we note that in schooling species such as roach, grouping with conspecifics may not only reduce predation risk, but also act to buffer against maladaptive behaviours expressed by individuals exposed to pharmaceutical pollutants.

## 5. Conclusions

A main limitation of many ecotoxicological studies is the lack of realism and the difficulty of extrapolating results obtained at an individual scale in controlled laboratory conditions to populations living in complex natural environments. Our study presents an integrative approach combining novel techniques to create an experiment in the field. We employed robust analytical methods that are widely used in animal behaviour [35,71] but have not been used in ecotoxicology, where they could reveal more ecologically relevant impacts of toxicants on wildlife, particularly in natural environments. Our results highlight ecologically important effects of psychotherapeutic drugs that enter aquatic ecosystems, such as shifts in fish habitat selection and behavioural states. Specifically, we showed that exposure to oxazepam and predation threat affected fish activity levels and selection of habitats with submerged vegetation. These complex behavioural responses could not be detected using traditional analytical methods. In a broader context, our findings call for more widespread inclusion of realistic experimental setups and advanced data analyses to examine the full environmental impact of pollutants on natural ecosystems.

## Data Availability

Raw data and datasets used for analysis as well as R scripts are available in the Dryad repository [72]. Supplementary material is available online [73].
